# Estimated dietary intake of polyphenols from cereal foods and associated lifestyle and demographic factors in the Melbourne Collaborative Cohort Study

**DOI:** 10.1038/s41598-023-35501-0

**Published:** 2023-05-26

**Authors:** Kristina Vingrys, Michael L. Mathai, Vasso Apostolopoulos, Julie K. Bassett, Maximilian de Courten, Lily Stojanovska, Lynne Millar, Graham G. Giles, Roger L. Milne, Allison M. Hodge, Andrew J. McAinch

**Affiliations:** 1 Institute for Health and Sport, Victoria University, PO Box 14428, Melbourne, VIC 8001 Australia; 2 VU First Year College ®, Victoria University, PO Box 14428, Melbourne, VIC 8001 Australia; 3 Australian Institute for Musculoskeletal Science (AIMSS), Victoria University, PO Box 14428, Melbourne, VIC 8001 Australia; 4 Cancer Epidemiology Division, Cancer Council Victoria, 615 St Kilda Rd, Melbourne, VIC 3004 Australia; 5 Mitchell Institute for Education and Health Policy, Victoria University, 300 Queen St, Melbourne, VIC Australia; 6 Department of Nutrition and Health, College of Medicine and Health Sciences, United Arab Emirates University, Al Ain, UAE; 7 Telethon Kids Institute, 15 Hospital Avenue, Nedlands, WA 6009 Australia; 8 Centre for Epidemiology and Biostatistics, Melbourne School of Population and Global Health, The University of Melbourne, Parkville, VIC Australia; 9 Precision Medicine, School of Clinical Sciences at Monash Health, Monash University, Clayton, VIC Australia

**Keywords:** Secondary metabolism, Nutrition, Epidemiology, Lifestyle modification

## Abstract

Cereal foods are consumed globally and are important sources of polyphenols with potential health benefits, yet dietary intakes are unclear. We aimed to calculate the dietary intakes of polyphenols from cereal foods in the Melbourne Collaborative Cohort Study (MCCS), and describe intakes by demographic and lifestyle factors. We estimated intakes of alkylresorcinols, lignans and phenolic acids in n = 39,892 eligible MCCS participants, using baseline dietary data (1990–1994) from a 121-item FFQ containing 17 cereal foods, matched to a polyphenol database developed from published literature and Phenol-Explorer Database. Intakes were estimated within groups according to lifestyle and demographic factors. The median (25th–75th percentile) intake of total polyphenols from cereal foods was 86.9 mg/day (51.4–155.8). The most consumed compounds were phenolic acids, with a median intake of 67.1 mg (39.5–118.8), followed by alkylresorcinols of 19.7 mg (10.8–34.6). Lignans made the smallest contribution of 0.50 mg (0.13–0.87). Higher polyphenol intakes were associated with higher relative socio-economic advantage and prudent lifestyles, including lower body mass index (BMI), non-smoking and higher physical activity scores. The findings based on polyphenol data specifically matched to the FFQ provide new information on intakes of cereal polyphenols, and how they might vary according to lifestyle and demographic factors.

## Introduction

Phenolic compounds (polyphenols) are phytochemicals found widely in plant-derived foods^1^ and contribute to the health benefits associated with plant foods, yet their nutritional role and mechanisms are still being investigated^2^. Grains and cereal foods are important sources of dietary polyphenols as staple foods in regional dietary patterns including in Australia^3^ and in Mediterranean countries such as Greece and Italy^4^, featuring foods such as bread, pasta, pizza, rice, oats and breakfast cereals^3–5^.

Polyphenols are broadly categorized as flavonoids and non-flavonoids^1^. Grains and cereal foods contain various polyphenol classes, notably the non-flavonoids; alkylresorcinols, lignans and phenolic acids, with flavonoids also reported in some varieties^6,7^. Wholegrains are richer sources of polyphenols than refined grains as polyphenol content is largely associated with the bran component^8–12^. Polyphenols may be mediators of associations between cereal foods and health outcomes, however reliable estimates of dietary intake are lacking, which is important to address as several polyphenols are exclusively or predominantly found in cereal foods^13,14^.

Understanding intake levels of polyphenols from grains and cereals in population studies may be useful to complement experimental studies and increase our understanding of relationships between dietary exposures and disease risk. Ferulic acid, a major phenolic acid in grains^13,14^, is associated with improved cardiovascular markers in humans^15,16^, inhibiting cancer cell proliferation^17,18^ and reducing renal damage in diabetes^19^. Alkylresorcinols, predominantly in rye and wheat, and avenanthramides, exclusive to oats^14^, have demonstrated anti-cancer properties^20,21^, antioxidant activity^22,23^ and may reduce cardiovascular and diabetes risks^24–27^. Grains are also sources of lignans^28^, phytoestrogenic compounds that may reduce cancer risk via estrogen receptors, antioxidant and anti-cancer mechanisms^29^.

However, despite their global consumption and potential health benefits, there are limited epidemiological data on cereal polyphenol intakes, and their associations with disease outcomes in human populations are unclear.

In order to investigate associations between cereal polyphenols and disease outcomes, data on polyphenol contents in foods are required; there are several approaches to obtain these^30–32^, Several cohort studies have assessed polyphenol intakes from total foods, often utilizing a single polyphenol data source such as the Phenol-Explorer Database (PED)^33–38^, which is a freely available dataset comprising over 500 polyphenols^7,39,40^ or the USDA flavonoid^41^ or isoflavone databases^42^. However, as food composition databases are often aggregated from multiple studies, specificity may be lost when applied to food frequency questionnaire (FFQ) items used in particular cohorts. Discrepancies in polyphenol analysis have also been found between databases^43^, including estimates of total aglycones from cereals and baked products reflecting results that were two times higher when estimated using PED compared with the USDA database^43^. Therefore, sourcing polyphenol data from a single database rather than utilizing multiple and specific sources may introduce intake estimation errors. Utilizing multiple data sources can also increase the accuracy of matching food items and their components whilst decreasing the likelihood of gaps in the final dataset. When creating a polyphenol dataset to use with available dietary intake data, it is also important to minimize missing values to increase the integrity and usability of the resultant polyphenol intake data^30,31^.

Determining the intakes of polyphenols from cereal foods in epidemiological studies is important to help understand their relationship with lifestyle and demographic variables and the potential mechanisms by which grains and cereal foods may contribute to disease risk. Presenting intakes across demographic/lifestyle variables is important when comparing intakes to other populations, and identifying associations with these variables will inform studies of associations between polyphenols and health outcomes. Therefore, this study aimed to estimate cereal polyphenol intakes in the Melbourne Collaborative Cohort Study (MCCS) and describe intakes across categories of lifestyle and demographic variables.

## Methods

### Study sample

The MCCS is a prospective population-based study, designed to investigate relationships between lifestyle related exposures, including diet, and chronic disease outcomes^44^. The MCCS includes 41,513 participants (24,469 females and 17,044 males) recruited between 1990 and 1994, with approximately 25% being migrants from southern Europe (primarily Italy and Greece)^44^. Most participants were identified from the state of Victoria’s voting register (Victorian Electoral Enrolment Register); enrolment to vote is mandatory for all Australian citizens over 18 years of age. At the time of cohort setup, the population of Melbourne was primarily of British descent but also included a large number of Italian and Greek migrants who were over-sampled to broaden the dietary intake, including the potential health benefits of the Mediterranean Diet. Most participants (99%) were aged between 40 and 69 years at recruitment and evenly distributed across 40–49, 50–59 and 60–69 age decades^44^. Data collection at baseline utilized a self-administered FFQ and interviewer-administered questionnaires for sociodemographic and lifestyle data^44^.

This study was conducted according to the guidelines laid down in the Declaration of Helsinki. All procedures involving research study participants were approved by the Cancer Council Victoria Human Research Ethics Committee (IEC9001) and Victoria University Human Research Ethics Committee (HRE 16-289) and participants provided informed consent^44^.

### Dietary assessment

Dietary intake data were collected at baseline using a 121-item FFQ designed specifically for the MCCS with cereal food item categories including breakfast cereals, breads, rice dishes, pasta and sweet and savoury baked items^45^. The list of included foods in the FFQ was based on weighed food records in 810 healthy, middle aged male and female volunteers of a similar demographic background to MCCS participants^45^ and was originally designed to capture habitual dietary intake of wide-range of foods and nutrients to prospectively investigate diet-disease relationships^44,45^.

The FFQ has been validated in several studies of bioactive compounds and micronutrients, including assessment of antioxidant intake (carotenoids: α-carotene, β-carotene, β-cryptoxanthin, lycopene and lutein/zeaxanthin) using plasma biomarkers^46^ and fatty acid intake using plasma phospholipid fatty acids^47^. Repeated application of an updated version of FFQ after 12 months has shown good repeatability for nutrients (n = 23) with ICC range for absolute nutrient intakes between 0.51 and 0.74 for Greek/Italian-born participants, and higher for Australian-born participants of 0.66 and 0.80^48^. Further details of the MCCS FFQ and reliability and validity studies are described elsewhere^44–46,48,49^.

### Procedure to create the polyphenol database

The methods to create the polyphenol database for the MCCS FFQ were adapted from published methods primarily from the EPIC polyphenol-food database^50,51^.

#### Foods and compounds for analysis

The MCCS FFQ includes 17 items within the ‘Cereal foods, cakes and biscuits’ group. All 17 of these items were included for this analysis, namely: wheatgerm; muesli; other breakfast cereals; rice, boiled (including brown rice); fried rice; mixed dishes with rice; white bread, rolls or toast; wholewheat or rye bread, rolls or toast; fruit bread; crackers or crispbreads; sweet biscuits; cakes or sweet pastries; puddings; pasta or noodles; pizza; dim sims or spring rolls; pies or savoury pastries.

The polyphenol compounds estimated (alkylresorcinols^11^, lignans^29^ and phenolic acids^52^, including avenanthramides^14^) included those commonly found in grains and cereal products included in the MCCS FFQ, which were mainly derived from wheat, oats, rice and rye. Compounds within these classes were prioritized based on their quantity, data available and use in previous studies. Further refinement occurred through omitting compounds with missing values, only 1–2 values^50^ or negligible values^53^. The final classes and compounds for analysis comprised: alkylresorcinols (5-*n*-Heneicosylresorcinol: C21:0; 5-*n*-Heptadecylresorcinol: C17:0; 5-*n*-Nonadecylresorcinol: C19:0; 5-*n*-Pentacosylresorcinol: C25:0; and 5-*n*-Tricosylresorcinol: C23:0), lignans (lariciresinol, matairesinol, pinoresinol and secoisolariciresinol), and phenolic acids, comprising ferulic acid and avenanthramides (Avenanthramide 2c, Avenanthramide 2f and Avenanthramide 2p).

#### Searching databases and published literature

Potential matches of polyphenol data with corresponding food items commenced with searching for items on PED v.3.6^7,54^, followed by published literature, using the common name of the food and synonyms. To evaluate the suitability of data from published literature, the criteria described for ‘critical evaluation of data’ by the PED were adapted^39^. Published literature was prioritized as a source of values to increase the specificity of the match to the food item as PED values were frequently averaged from data pertaining to several foods, some of which were not relevant to the items in the present study. Multiple sources were used to reduce the likelihood of discrepancies that may occur when using single databases to source polyphenol data^43^. As some polyphenols are glycosylated or esterified to the matrix (such as phenolic acids and lignans), these compounds need to be released and solubilized for analysis and therefore values from chromatography after hydrolysis were used where available^39^.

No published datasets were found for lignans, alkylresorcinols or phenolic acids in Australian products, but datasets were located for similar cereals and cereal products mainly from Europe, Canada and the UK^11,14,55–63^ (see Additional File 1). These data met the inclusion criteria for this study, values contained were all derived using chromatography, and have previously been used by other published studies developing ad-hoc databases^53,64–66^. Values for individual alkylresorcinols were derived from percentages of total alkylresorcinols reported in the literature^11,14,58,60^.

#### Matching food items to sources

The matching of foods to polyphenol data was adapted from Knaze et al.^50^ with minor modifications. Polyphenol data were coded for the number of potential matches (no match, "single" match, "multiple" match or "complex" food to be deconstructed), and number of polyphenol items and match quality (“none”, “exact”, “similar” or “missing”). Codes were also applied for data source (specific database or published dataset) and derivation (how a value was calculated or imputed)^30,67^. For example, lariciresinol content in rice-based ready-to-eat breakfast cereal was imputed from boiled white rice^55^, representing rice as the same food but in different forms (puffed or boiled). Similarly, lariciresinol in crackers and crispbreads was imputed from data for whole grain wheat and rye bread^55^, representing values from similar but different foods.

Matches took into account raw, cooked or processed state where possible^50^. Where multiple matches were returned the most appropriate food item was selected or food items were aggregated and the mean of values used^68^. A ‘missing’ match was one where the food was expected to contain polyphenols but there was insufficient data, consequently alternatives were sought or values from other literature explored. Agreement for matching the items was reached by regular discussion between the research team during the data extraction process. All FFQ items were able to be matched to polyphenol data for exact or similar foods.

#### Missing and zero values

A ‘missing’ match was one where the food was expected to contain polyphenols but there was insufficient data, consequently alternatives were sought or values from other literature explored. Values that were not initially matched directly from PED were flagged as missing values and other sources were used to fill in the data points. Zero values and blank values were frequent occurrences in the first matching stage. Logical zeros were applied where it was reasonably assumed that the item contained little or none of a particular polyphenol^30,50^. Data were imputed from the literature if missing or an unreasonable zero in PED rather than leave blanks^31,69^.

#### Deconstruction of complex foods

Many of the MCCS items were analyzed as complex foods, defined in this study as a food or beverage item comprising multiple ingredients^50,70^. Where data was available for complex foods as a single item this was used. Otherwise, complex foods were deconstructed into their constituent components with proportions from a recipe, listing each ingredient separately and calculating the polyphenol content of each ingredient to derive the totals in the food item. To derive ingredients and proportions, food items were searched in a local Australian food composition database (Australian Food Composition Database—Release 1 (AFCD-1)^71^ or relevant internet recipes from Australian recipe websites or cookbooks, and averaged, similar to the methods described by Knaze et al.^50^.

Once the complex items were deconstructed, the major cereal or grain component and proportion were used to estimate the polyphenol content from the principal ingredient (See Additional file 2). Constituent ingredients were searched individually for polyphenol content and then aggregated with the combined proportions equating to 100%^50,70^. Proportions of ingredients as proxies have been estimated from recipe deconstruction of mixed dishes in other studies^33,35,50^.

Where proxies were used for missing values, manufacturer’s percentages were applied, such as 97% wholegrain wheat flour for wheat breakfast biscuits (see Additional File 2). ‘Rice boiled, including brown rice’ was adjusted to correspond to 90% white rice and 10% brown rice in line with adult consumption patterns reported in Australia^72^ and the UK^73^. ’Wholewheat or rye bread, rolls or toast’ was adjusted to reflect 80% wholewheat (wholegrain, wholewheat or multigrain) and 20% rye, aligned with intake proportions calculated from dietary records collected from a subsample of MCCS participants at a follow-up survey (2003 to 2007)^44^. Crackers or crispbreads utilized bread as a proxy for some values and intake proportions of 90% and 10% were applied to wheat and rye products respectively. Where we encountered missing details in the FFQ, for example the item breakfast cereals did not distinguish between cereal types, we made some evidence-based assumptions about representative cereals consumed at that time and averaged those to determine the polyphenol content for ‘breakfast cereals’. Further details are reported in Additional File 2.

#### Utilization, classification and calculating the polyphenol data

Data were imported to the MCCS database in units of mg/100 g to align with the PED database. Items were recorded in an Excel table, with polyphenol data extracted from the PED then checked and supplemented with available literature.

Where possible, values were multiplied by a retention factor to account for losses associated with food processing^40,50^. A retention factor of 1 was applied to avenanthramides, lignans and alkylresorcinols to reflect the absence of retention factor data in PED^7^, and minimal losses reported for lignans and alkylresorcinols^11,55^. Specific retention factors for ferulic acid were applied for food and processing methods^7,40^ (See Additional File 3).

### Calculation of intakes and statistical analysis

**Figure 1:**
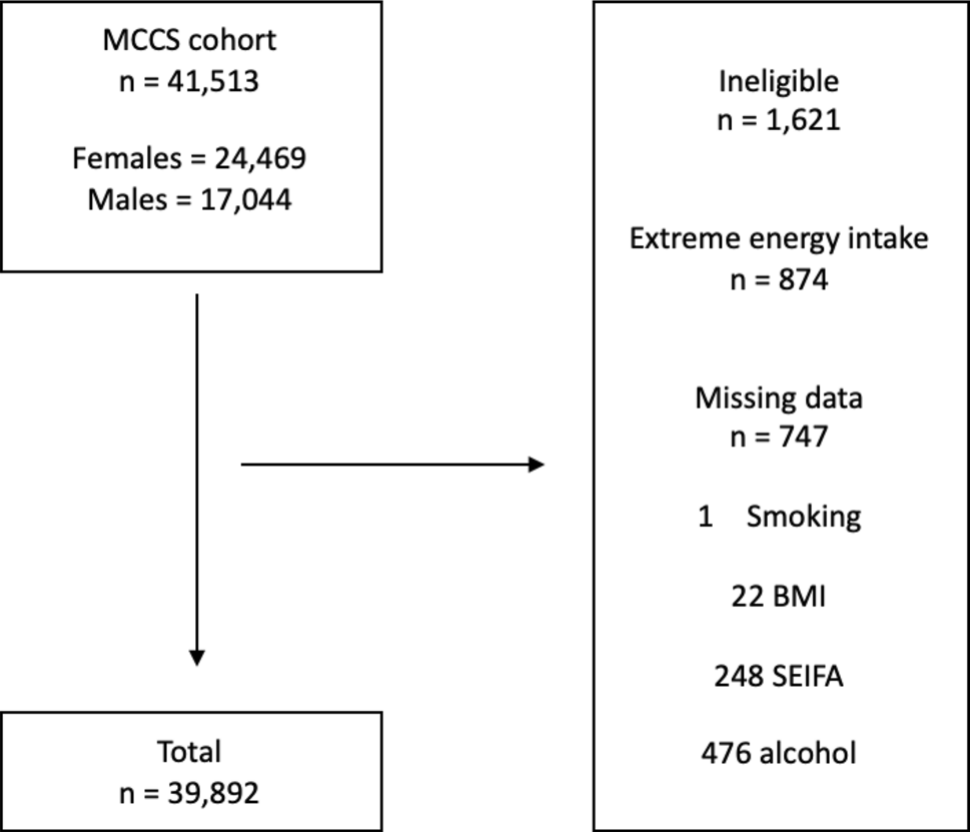
Flow diagram summarizing inclusion of participants for the data analysis. *BMI* body mass index (kg/m^2^), *SEIFA* Quintiles of Index of Relative Socioeconomic Disadvantage, *MCCS* Melbourne Collaborative Cohort Study.

We excluded participants that were missing dietary data (exposure) or other analysis variables (n = 747) and those who reported extreme energy intakes (highest or lowest 1% of the sex-specific distributions (n = 874) (Fig. 1).

To calculate the polyphenol intake of MCCS participants the reported frequencies of consumption for individual food items (9 categories from never or < 1/month to 6 +/day) were converted to daily equivalents and multiplied by sex-specific standard portion sizes to estimate the amount of each item consumed. This amount was then multiplied by the polyphenol content (mg/100 g)^74^. Total cereal polyphenols were estimated as the sum of alkylresorcinols, lignans and phenolic acids.

Overall intakes of cereal polyphenols are presented, as well as intakes according to sub-groups of socio-demographic factors: sex (female, male), age (≤ 49, 50–59, ≥ 60 years), body mass index (BMI in kg/m^2^, < 25, 25–30, > 30), country of birth (Australia/New Zealand, UK/Malta, Italy, Greece), smoking status (never, former, current), alcohol drinking status (lifetime abstainers, ex-drinkers, < 20 g/day, 20–39 g/day, ≥ 40 g/day), and physical activity score, where frequency responses (0 (none), 1.5 (one or two times per week), and 4 (≥ 3 times per week) for walking, less vigorous and vigorous activity were summed after assigning twice the weight to vigorous activity (the overall score ranged from 0 to 16 and was divided into roughly equal groups using the cut points: 0, > 0 to < 4, 4 to < 6, ≥ 6). Socio-economic position was categorized using Index of Relative Socioeconomic Disadvantage (SEIFA) in quintiles, Q1: most disadvantaged–Q5 least disadvantaged). SEIFA is an area- based multidimensional social and economic classification index that describes the shared socio-economic status, in terms of relative advantage and disadvantage, including education and other variables^75^.

Histograms of the distribution of total polyphenol data suggested a skewed distribution, therefore median values (25th–75th percentile) were reported. Mean ± SD values are shown for comparative purposes as recommended when reporting observational studies in epidemiology^76^. Differences in polyphenol intakes across demographic and lifestyle categories were evaluated using the Mann–Whitney *U* test and Kruskal–Wallis test as appropriate. A p-value of < 0.05 was considered statistically significant. Stata MP 16.1 statistical software was used for analysis^77^. The STROBE-nut checklist was used for reporting the study^78^ (see Additional File 4).

### Ethics approval and consent to participate

MCCS participants provided informed consent^31^ and the study was approved by Cancer Council Victoria Human Research Ethics Committee (IEC9001) and Victoria University Human Research Ethics Committee (HRE 16-289).

## Results

### Polyphenol database of MCCS cereal foods

A polyphenol database for 17 cereal food items listed in the MCCS FFQ was developed, comprising 13 phenolic compounds: five alkylresorcinols (5-*n*-Heneicosylresorcinol: C21:0; 5-*n*-Heptadecylresorcinol: C17:0; 5-*n*-Nonadecylresorcinol: C19:0; 5-*n*-Pentacosylresorcinol: C25:0; and 5-*n*-Tricosylresorcinol: C23:0), four lignans (lariciresinol, matairesinol, pinoresinol and secoisolariciresinol), and four phenolic acids, comprising one hydroxycinnamic acid (ferulic acid) and three avenanthramides (Avenanthramide 2c, Avenanthramide 2f and Avenanthramide 2p).

The source of values were mainly datasets in published literature (93%). Of those values, complex foods made up 36% of items that were calculated/imputed by deconstruction, whereas, logical zeros represented the smallest percentage (1%) of data calculations/imputations.

### Included participants

Of the 41,513 MCCS participants, 1621 were excluded, leaving 39,892 available for the final analysis (Fig. 1). Excluded participants reported energy intakes in the highest or lowest 1% of the sex specific distributions (n = 874) or were missing data (n = 747: n = 1 smoking, n = 22 BMI, n = 248 SEIFA, n = 476 alcohol). The sample comprised more females (59%) than males (41%) (Table 1). The mean (± SD) age was 55 (± 9) years and mean BMI was 27 kg/m^2^ (± 4).

**Table:** Dietary intakes of cereal polyphenols by demographic and lifestyle characteristics in the MCCS (n = 39,892).

	*n*	%	Total polyphenols^a^ (mg/day)		Lignans^b^ (mg/day)		Alkylresorcinols^c^ (mg/day)		Phenolic acids^d^ (mg/day)	
			Median	25th–75th percentile	Median	25th–75th percentile	Median	25th–75th percentile	Median	25th–75th percentile
Demographics										
Sex										
Female	23,680	59	84.5	51.6–163.4	0.53	0.15–0.91	18.9	11.4–33.8	65.3	39.0–128.3
Male	16,212	41	90.7	51.4–151.9	0.41	0.12–0.82	20.6	10.2–35.7	69.9	40.6–115.6
p-value			< 0.001		0.181		< 0.001		< 0.001	
Age, years										
≤ 49	12,638	32	81.3	48.6–132.5	0.43	0.14–0.76	17.8	9.7–31.5	62.7	37.8–102.0
50–59	13,021	33	85.1	49.5–146.8	0.49	0.13–0.84	19.1	10.2–33.5	65.7	38.4–112.4
≥ 60	14,233	36	96.6	56.5–178.3	0.59	0.14–1.45	22.2	13.4–38.6	73.7	42.7–140.2
p-value			< 0.001		< 0.001		< 0.001		< 0.001	
Body mass index (kg/m^2^)										
< 25	14,458	36	96.2	60.6–173.1	0.60	0.24–1.44	21.7	13.7–36.6	74.0	46.0–135.9
25–30	17,242	43	85.5	49.9–148.4	0.47	0.12–0.83	19.6	10.3–34.1	65.8	38.5–113.2
> 30	8,192	21	75.5	40.2–130.3	0.34	0.10–0.74	16.6	7.7–31.2	57.8	32.1–99.5
p-value			< 0.001		< 0.001		< 0.001		< 0.001	
Country of birth, *n* (%)										
Australia/New Zealand	27,522	69	95.0	60.7–170.5	0.58	0.18–1.42	22.0	14.1–36.8	72.4	45.6–133.5
United Kingdom/Malta	2,928	7	94.6	59.2–168.8	0.58	0.20–1.02	21.1	13.2–36.5	72.8	45.2–131.6
Italy	5,176	13	61.4	33.9–103.8	0.17	0.08–0.62	13.2	4.9–21.6	47.9	28.3–81.8
Greece	4,266	11	62.3	25.2–106.8	0.29	0.07–0.71	12.1	3.7–21.6	49.2	21.2–84.2
p-value			< 0.001		< 0.001		< 0.001		< 0.001	
SEIFA^e^, *n* (%)										
Q1	7,243	18	76.9	42.4–133.6	0.35	0.10–0.75	17.4	8.2–32.3	58.8	33.0–101.8
Q2	8,379	21	80.3	44.7–141.1	0.39	0.11–0.78	18.1	8.8–33.3	61.7	35.0–107.4
Q3	6,279	16	84.6	49.5–152.7	0.49	0.12–0.87	19.2	10.3–34.2	65.4	38.0–117.0
Q4	7,381	19	92.8	57.0–167.8	0.57	0.17–1.00	21.0	12.5–35.9	71.4	43.4–131.3
Q5	10,610	27	97.1	63.1–169.1	0.60	0.27–1.02	21.9	13.9–36.3	74.7	48.2–132.3
p-value			< 0.001		< 0.001		< 0.001		< 0.001	
Lifestyle behaviors										
Smoking status, *n* (%)										
Never	23,033	58	89.1	54.3–163.6	0.55	0.15–0.95	20.1	11.9–34.8	68.7	41.3–127.6
Former	12,478	31	90.8	55.1–162.8	0.54	0.14–0.95	20.6	11.7–35.9	70.2	42.2–126.2
Current	4,381	11	65.1	32.8–108.4	0.27	0.08–0.65	14.7	5.5–25.6	49.5	26.6–82.9
p-value			< 0.001		< 0.001		< 0.001		< 0.001	
Alcohol drinking status, *n* (%)										
Lifetime abstainers	11,461	29	82.0	46.4–153.0	0.48	0.11–0.86	18.5	9.5–33.7	63.1	35.3–118.3
Ex-drinkers	4,192	11	86.0	51.0–151.6	0.48	0.13–0.85	19.5	10.3–34.6	66.2	39.6–115.7
Low intake (< 20 g/day)	16,707	42	91.6	57.5–165.7	0.56	0.16–0.97	20.9	12.7–35.4	70.6	44.0–129.4
Medium intake (20–39 g/day)	4,733	12	88.6	52.7–152.1	0.54	0.14–0.86	19.8	10.5–34.5	68.9	41.3–116.0
High intake (≥ 40 g/day)	2,799	7	77.6	40.4–127.2	0.36	0.10–0.75	17.0	7.6–30.2	59.1	32.5–97.8
p-value			< 0.001		< 0.001		< 0.001		< 0.001	
Physical activity score, *n* (%)										
Zero	8,803	22	73.1	37.7–124.6	0.31	0.09–0.73	16.3	6.8–29.5	55.7	30.2–95.4
> 0 to < 4	8,031	20	84.2	50.3–147.6	0.48	0.13–0.84	19.1	10.3–33.9	65.0	38.5–112.7
4 to < 6	14,194	36	89.6	53.8–163.2	0.55	0.14–0.94	20.2	11.8–35.3	69.2	41.1–126.4
≥ 6	8,864	22	100.1	65.9–176.6	0.60	0.28–1.44	22.7	14.4–37.4	77.3	50.1–138.8
p-value			< 0.001		< 0.001		< 0.001		< 0.001	

Differences across categories were evaluated using the Mann–Whitney U test or the Kruskal–Wallis test as appropriate. Percentages may not add to 100 dues to rounding.

^a^Sum of alkylresorcinols, lignans and phenolic acids from cereal foods.

^b^Sum of lariciresinol, matairesinol, pinoresinol and secoisolariciresinol from cereal foods.

^c^Sum of 5-*n*-Heneicosylresorcinol (C21:0), 5-*n*-Heptadecylresorcinol (C17:0), 5-*n*-Nonadecylresorcinol (C19:0), 5-*n*-Pentacosylresorcinol (C25:0) and 5-*n*-Tricosylresorcinol (C23:0) from cereal foods.

^d^Sum of ferulic acid and avenanthramides (Avenanthramide 2c, Avenanthramide 2f. and Avenanthramide 2p) from cereal foods.

^e^SEIFA = Quintiles of Index of Relative Socioeconomic Disadvantage: Q1 (most disadvantaged); Q5 (least disadvantaged).

*MCCS* Melbourne Collaborative Cohort Study.

Most participants (64%) had a BMI categorized as either overweight (43% BMI 25–30 kg/m^2^) or obese (21% BMI > 30 kg/m^2^) and were of Anglo-Celtic origin (76%), primarily born in either Australia or New Zealand. Nearly one-quarter of participants were of Southern-European origin (13% Italy and 11% Greece) (Table 1).

### Cereal polyphenol intakes by polyphenol class and compound

The median intake of total polyphenols from cereal foods was 86.9 mg/day (51.4–155.8) (Table 2). Phenolic acids, mainly ferulic acid, were the most consumed compounds. Ferulic acid intake contributed 77% of total cereal polyphenols and 99% of total phenolic acids were ferulic acid. The median intake of ferulic acid for the cohort was 66.8 mg/day (39.3–118.1). Alkylresorcinols were the second highest contributor to total cereal polyphenol intakes, with a median intake of 19.7 mg (10.8–34.6). The main compound contributing to alkylresorcinol intakes was 5-*n*-Heneicosylresorcinol (C:21, 8.3 mg (4.3–12.9)). Lignans provided the smallest contribution to intakes of cereal polyphenols in this cohort, with a median intake of 0.50 mg (0.13–0.87), mostly as secoisolariciresinol.

**Table 2:** Dietary intakes of energy, dietary fiber and cereal polyphenols in the MCCS (n = 39,892).

	Median	25th–75th percentile	Mean	SD
Energy intake (kJ/day)	8698	6936–10,875	9160	3109
Dietary fiber intake (g/day)	29.2	22.7–37.1	30.9	12.0
Total cereal polyphenol intake (mg/day)^a^	86.9	51.4–155.8	113.1	88.5
Lignans (mg/day)^b^	0.50	0.13–0.87	0.71	0.73
Lariciresinol	0.09	0.05–0.17	0.11	0.09
Matairesinol	0.01	0.00–0.01	0.01	0.01
Pinoresinol	0.10	0.03–0.24	0.14	0.14
Secoisolariciresinol	0.32	0.05–0.50	0.45	0.51
Alkylresorcinols (mg/day)^c^	19.7	10.8–34.6	24.9	19.8
5-*n*-Heptadecylresorcinol (C17:0)	2.2	0.8–4.0	3.0	2.9
5-*n*-Nonadecylresorcinol (C19:0)	6.4	3.6–11.8	8.4	6.8
5-*n*-Heneicosylresorcinol (C21:0)	8.3	4.3–12.9	9.6	7.3
5-*n*-Tricosylresorcinol (C23:0)	2.2	1.3–3.6	2.7	2.0
5-*n*-Pentacosylresorcinol (C25:0)	0.9	0.5–1.8	1.3	1.1
Phenolic acids (mg/day)^d^	67.1	39.5–118.8	87.5	68.7
Ferulic acid	66.8	39.3–118.1	87.2	68.6
Avenanthramides^e^	0.17	0.03–0.24	0.28	0.43
Avenanthramide 2c	0.05	0.01–0.07	0.09	0.14
Avenanthramide 2f	0.07	0.01–0.10	0.11	0.16
Avenanthramide 2p	0.05	0.01–0.08	0.08	0.12

^a^Sum of alkylresorcinols, lignans and phenolic acids from cereal foods.

^b^Sum of lariciresinol, matairesinol, pinoresinol and secoisolariciresinol from cereal foods.

^c^Sum of 5-*n*-Heneicosylresorcinol (C21:0), 5-*n*-Heptadecylresorcinol (C17:0), 5-*n*-Nonadecylresorcinol (C19:0), 5-*n*-Pentacosylresorcinol (C25:0) and 5-*n*-Tricosylresorcinol (C23:0) from cereal foods.

^d^Sum of ferulic acid and avenanthramides (Avenanthramide 2c, Avenanthramide 2f and Avenanthramide 2p) from cereal foods.

^e^Sum of Avenanthramide 2c, Avenanthramide 2f and Avenanthramide 2p.

*MCCS* Melbourne Collaborative Cohort Study.

### Cereal polyphenol intakes by demographic and lifestyle characteristics

Participants with higher intakes of cereal polyphenols were generally male, older (≥ 60 years), with lower BMI, born in Australia/New Zealand, less socio-economically disadvantaged, non-smokers and had higher physical activity scores. Higher intakes for men was associated with higher intakes of alkylresorcinols (p < 0.001) and phenolic acids (p < 0.001).

## Discussion

We developed a cereal polyphenol database from multiple sources and estimated dietary intakes for an ethnically diverse cohort of 39,892 MCCS participants. Our findings suggest that participants with indicators of a prudent lifestyle (lower BMI, higher physical activity scores, non-smokers) and males consumed more polyphenols from cereal foods than those with less healthy lifestyles and females. Participants with higher relative socio-economic advantage (higher SEIFA categories) also consumed more polyphenols from cereal foods. Participants from Australia/New Zealand had higher cereal polyphenol intakes compared with participants from Italy and Greece. The main cereal-derived polyphenols consumed were phenolic acids, mainly ferulic acid.

Similar to our study, total polyphenol intakes, from all foods, were associated with higher physical activity in a Polish cohort^33^, but not in other European cohorts^37^. Higher education levels were also found to be associated with higher total polyphenol intakes from all foods in some cohorts^33,35,37^, but an inverse association has been reported by others^74^. In contrast to our study, current smoking was found to be associated with higher polyphenol intakes from all foods in other studies^33,37,74^. This may be attributed to intake of polyphenols from coffee consumption which is associated with smoking^33,37,74^.

Males had higher median intakes of total cereal polyphenols of 90.7 (51.4–151.9) mg/day compared with females (84.5, 51.6–163.4) mg/day, which was associated with men having higher intakes of alkylresorcinols and phenolic acids. Higher energy intakes in males may have contributed to our observations but weren’t assessed in this study. Sex differences in intakes of total polyphenols are varied in other studies, with both higher^35^ and lower intakes reported in males^33^ and no differences between males and females in other cohorts^37,74^.

The median (25th–75th percentile) intake of polyphenols from cereal foods in the MCCS was 86.9 mg/day (51.4–155.8), and the mean intake was 113.1 mg/day (± 88.5). Lower values of polyphenols from cereal foods were reported in the Polish arm of the Health, Alcohol and Psychosocial factors in Eastern Europe (HAPIEE) study, with a mean of 38.9 mg/day (± 29.8) from cereals^33^. The higher values estimated for the MCCS may be partly attributed to estimation methods, where we used multiple sources to estimate intakes, thus reducing the risk of underestimation by minimizing the use of zero values if appropriate polyphenol data could be obtained. Estimated higher values of total polyphenols from cereal foods, 793–1087 mg/day, were associated with a Mediterranean diet in a Spanish study^79^; the higher values calculated may be explained by differences in dietary factors and estimation methods^79^. The Spanish study also suggested that cereals contributed the largest proportion of polyphenols to the diet due to the large amounts consumed, despite having the lowest content of total polyphenols of the foods analyzed^79^.

Whilst the intake of cereal foods may vary between cohorts, in Australian adults in the 1995 National Nutrition Survey, cereals and cereal products contribute approximately 20% of total energy intake^80^ therefore dietary polyphenol intake from cereals is likely to contribute a significant amount to total polyphenol intake. However, wholegrain consumption has decreased over time globally^81,82^, potentially reducing any health benefits from polyphenols that may be associated with these foods^83^.

Phenolic acids (ferulic acid), were the main cereal-derived polyphenols consumed in the MCCS. The median ferulic acid intake in the MCCS was 66.8 mg/day (39.3–118.1), and mean intake was 87.2 mg/day (± 68.6). Grains and cereal foods are recognized sources of phenolic acids, particularly ferulic acid^37,52,84,85^. Lower intakes of ferulic acid from all foods were reported from EPIC of mean 38 mg/day (± 0.3) where cereal foods were the top 3 contributors; from the Polish arm of the HAPIEE cohort and SU.VI.MAX cohort; mean intakes were 43.9 mg/day (± 33.7)^33^ and 8.3 mg/day (± 4.1)^35^ respectively, of which flour contributed 7%^33^ and refined flour products contributed 78%^35^.

Alkylresorcinols are also one of the most prevalent phenolic compounds in grains^8–11^, found primarily in wholegrain wheat and rye^11^. The estimated median intake of alkylresorcinols in the present study was 19.7 mg/day (10.8–34.6), and the mean alkylresorcinol intake was 24.9 mg/day (± 19.8), comprising predominantly 5-n-Heneicosylresorcinol (C21:0) followed by 5-n-Nonadecylresorcinol (C19:0). Consistent with the MCCS, 5-Heneicosylresorcinol and 5-Nonadecylresorcinol were prevalent compounds in bread and cereal foods consumed in both the EPIC cohort^37^ and in the Polish Arm of the HAPIEE cohort^33^. In the HAPIEE cohort, the mean intake of alkylphenols (alkylresorcinols) from total foods was estimated at 26.1 mg/day (± 32.1)^33^, however these appear to be exclusively associated with cereal foods thus similar to intakes in the MCCS.

Cereals and cereal products made from rye, wheat, oats and corn are also sources of dietary lignans^29^. In the MCCS, lignans contributed the smallest proportion of polyphenols from cereal foods, with an estimated median intake of 0.50 mg (0.13–0.87), and mean intake of 0.71 mg (± 0.73). Cereals were found to be key contributors to dietary lignan intakes in several studies in Denmark, Finland, Sweden, Italy and the United Kingdom^65^, attributable to the large quantities of cereals consumed, despite the comparatively low lignan content relative to foods such as sesame and flax seeds^79,83^. In the EPIC cohorts, the non-Mediterranean countries appeared to have the highest contribution of lignans from cereal products at approximately 2.2 mg/day (24%) based on a maximum total lignan intake of 9.1 mg/day^37^. Conversely, a relatively low mean intake of lignans was estimated in the Polish arm of the HAPIEE cohort, with mean intake of 0.1 mg/day (± 0.1)^33^. Relatively low intakes of lignans from total diets in other Australian studies^86,87^ are also in agreement with the low intakes of lignans from cereal foods compared with other phenolic compounds in the MCCS.

Avenanthramides specific to oats, are also minor but notable phenolic compounds in grains^14^. In the MCCS, avenanthramides contributed only 0.17 mg (0.03–0.24) to total phenolic acid intakes. Estimated avenanthramide intakes from other studies ranged from 0.3 to 2.1 mg^62^, which is higher than the median intake in the MCCS. The lower values for the MCCS may be explained by the low intake of oat-based cereals, with only approximately 15% of participants reporting consuming muesli and approximately 30% of participants consuming porridge at least once per week in winter.

Our results need to be considered in context of some limitations. Firstly, our study was limited to cereal polyphenols, to identify potential protective mechanisms of cereal foods and disease outcomes in future studies. The focus on selected polyphenols may have under-estimated the total polyphenols in cereal foods, but our findings were generally in line with other studies, suggesting that contributions from other compounds would be minor^52^. The MCCS cohort may not be representative of the general population as participants tend to be healthier, due to possible self-selection bias of healthy volunteers in research studies^88,89^. The MCCS also includes a greater proportion of Italian and Greek participants compared with the general population at the time of survey^44^.

Assessment of dietary intake was based on a 121-item FFQ with the potential for various reporting errors and biases common to all subjective dietary assessment^90–92^. Dietary intakes also may have changed over time and may not reflect current intakes. Using baseline cohort FFQs in any longitudinal cohort study analyzing dietary intake and disease incidence will be subject to this issue and the difference between current and retrospective diet histories can vary across cohorts^93^ and subgroups such as by sex^93^. The MCCS FFQ may not perform as well in assessing the intakes of southern European-born participants compared with Australian-born participants^48^, which may account for polyphenol intakes lower than expected in southern European-born participants. The FFQ was not validated for polyphenol intake and not specifically designed to assess polyphenols and cereal foods, however 17 items were included in the FFQ, which is more than for other studies^79^ and though not exhaustive was unlikely to exclude any major dietary cereal sources of polyphenols. The FFQ also did not contain details for all cereal items within each category, particularly pooled categories such as ‘breakfast cereals’, however we were able to apply methodology to miminize missing any key cereals and polyphenols consumed.

Despite the MCCS’ FFQ limitations, it has been used as a dietary assessment tool to show associations between diet and disease outcomes in this cohort^45,94–97^. Strengths of the present study include the large sample size and inclusion of participants from Southern-European origin which broadens the range of intakes. Utilizing multiple data sources to derive polyphenol data increased the specificity and improved the accuracy of the matches in the final data set^30,43^. This study’s focus on cereal food polyphenols has provided new information about the key cereal polyphenols consumed and socio-demographic associations with intakes in the MCCS, that may be useful for exploring diet-disease relationships relating to grain and cereal foods in future studies.

## Conclusion

We used a purpose-built grains and cereal polyphenol database to quantify the intakes of polyphenol classes and compounds from grains and cereal foods in the MCCS, and estimated intakes by demographic and lifestyle factors. This differs from other studies of polyphenol intake as it reports on polyphenol intakes specifically associated with grains and cereal foods; however, the estimated intakes and associations with prudent lifestyle indicators are somewhat comparable with those of other studies, supporting our findings.

The results fill a gap in data for dietary intakes of non-flavonoid compounds, namely alkylresorcinols, phenolic acids and lignans, particularly in Australia. Further research in cereal-derived phenolic compounds in populations will help clarify the role that bioactive compounds may play in diet-disease relationships, and the intake estimates may be utilized to investigate disease risks associated with these foods and compounds in the MCCS. The detailed methodology will also be useful to adapt for other food groups and cohorts when investigating polyphenols and disease risk.

## Supplementary Information

.

## Data Availability

The data are not publicly available due to protection of research participant privacy/consent. Enquiries regarding data access should be directed in the first instance to the corresponding author. Further details about using MCCS data can be found at: https://www.pedigree.org.au/default.aspx.

## Abbreviations

AFCD-1: Australian Food Composition Database - Release 1
BMI: Body mass index
EPIC: European Prospective Investigation into Cancer and Nutrition
FFQ: Food frequency questionnaire
HAPIEE: Health, Alcohol and Psychosocial Factors in Eastern Europe
MCCS: Melbourne Collaborative Cohort Study
PED: Phenol-Explorer Database
SEIFA: Socio-Economic Indexes for Areas (Index of Relative Socioeconomic Disadvantage)
STROBE: Strengthening the Reporting of Observational Sudies in Epidemiology
SU.VI.MAX: SUpplementation en VItamines et Mineraux AntioXydants
