# Supplementary material for: Estimated dietary intake of polyphenols from cereal foods and associated lifestyle and demographic factors in the Melbourne Collaborative Cohort Study
Source: Sci Rep. 2023 May 26;13:8556. doi: 10.1038/s41598-023-35501-0 (PMC10220042; doi:10.1038/s41598-023-35501-0)
Supplement: Supplementary file 1 — . [file 41598_2023_35501_MOESM1_ESM.pdf]

Estimated dietary intake of polyphenols from cereal foods and associated lifestyle and demographic factors in the Melbourne Collaborative Cohort Study

Kristina Vingrys[a,b], Michael L. Mathai[a], Vasso Apostolopoulos[a,c], Julie K. Bassett[d], Maximilian de Courten[a,e], Lily Stojanovska[a,f], Lynne Millar[a,g], Graham G. Giles[d,h,i], Roger L. Milne[d,h,i], Allison M. Hodge,[d,h,*], Andrew J. McAinch[a,c*]

[a] Institute for Health and Sport, Victoria University, PO Box 14428, Melbourne, VIC, 8001, Australia.

[b] VU First Year College ®, Victoria University, PO Box 14428, Melbourne, VIC, 8001, Australia.

[c] Australian Institute for Musculoskeletal Science (AIMSS), Victoria University, PO Box 14428, Melbourne, VIC 8001, Australia.

[d] Cancer Epidemiology Division, Cancer Council Victoria, 615 St Kilda Rd, Melbourne VIC 3004, Australia.

[e] Mitchell Institute for Education and Health Policy, Victoria University, 300 Queen St, Melbourne VIC, Australia.

[f] Department of Nutrition and Health, College of Medicine and Health Sciences, United Arab Emirates University, Al Ain, UAE.

[g] Telethon Kids Institute, 15 Hospital Avenue, Nedlands, WA, 6009, Australia.

[h] Centre for Epidemiology and Biostatistics, Melbourne School of Population and Global Health, The University of Melbourne, Parkville, VIC, Australia.

[i] Precision Medicine, School of Clinical Sciences at Monash Health, Monash University, Clayton, VIC, Australia.

*Joint Senior Authors

Corresponding Author

Dr. Kristina Vingrys

Institute for Health and Sport, Victoria University, PO Box 14428, Melbourne, VIC 8001, Australia.

Phone: 613 9919 5339 Email: kris.vingrys@vu.edu.au

Additional file 1

Summary of datasets and published literature used to create the Cereal Polyphenol Database for the present study

Origin	Data used	Reference
**Phenol Explorer Database version 3.6 Various sources**	Used for preliminary search to match all foods and their polyphenols, retention factors and guidelines for inclusion of other literature as described in 'critical evaluation of data'.	(1) Available at: http://phenol-explorer.eu/
**Netherlands**	Lariciresinol<br>Matairesinol<br>Pinoresinol<br>Secoisolariciresinol	(2)
**UK**	Matairesinol<br>Secoisolariciresinol	(3)
**Canada**	Lariciresinol<br>Matairesinol<br>Pinoresinol<br>Secoisolariciresinol	(4)
**US, Finland, UK**	Secoisolariciresinol<br>Matairesinol	(5)
**Sweden**	5-*n*-Heptadecylresorcinol<br>5-*n*-Nonadecylresorcinol<br>5-*n*-Heneicosylresorcinol<br>5-*n*-Tricosylresorcinol	(6-8)

Origin	Data used	Reference
	5-*n*-Pentacosylresorcinol	
**UK**	Avenanthramide-C<br>Avenanthramide-A<br>Avenanthramide-B	(9)
**Sweden, UK, US**	Avenanthramide 2p<br>Avenanthramide 2f<br>Avenanthramide 2c	(10)
**European**	Avenanthramide 2c<br>Avenanthramide 2p<br>Avenanthramide 2f	(11)
**Finland**	5-Heptadecylresorcinol<br>5-Nonadecylresorcinol<br>5-Heneicosylresorcinol<br>5-Tricosylresorcinol<br>5-Pentacosylresorcinol<br>Ferulic acid<br>Avenanthramide 2c<br>Avenanthramide 2p<br>Avenanthramide 2f	(12)

UK = United Kingdom; US = United States.

REFERENCES

1. Neveu V, Perez-Jimenez J, Vos F, Crespy V, du Chaffaut L, Mennen L, et al. Phenol-Explorer: an online comprehensive database on polyphenol contents in foods. Database-Oxford. 2010;2010(bap024).
2. Milder IEJ, Arts ICW, Putte Bvd, Venema DP, Hollman PCH. Lignan contents of Dutch plant foods: a database including lariciresinol, pinoresinol, secoisolariciresinol and matairesinol. British Journal of Nutrition. 2005;93(3):393-402.
3. Kuhnle GGC, Dell'Aquila C, Aspinall SM, Runswick SA, Mulligan AA, Bingham SA. Phytoestrogen content of cereals and cereal-based foods consumed in the UK. Nutr Cancer. 2009;61(3):302-9.
4. Thompson LU, Boucher BA, Liu Z, Cotterchio M, Kreiger N. Phytoestrogen content of foods consumed in Canada, including isoflavones, lignans, and coumestan. Nutrition & Cancer. 2006;54(2):184-201.
5. Mazur W, Fotsis T, Wähälä K, Ojala S, Salakka A, Adlercreutz H. Isotope dilution gas chromatographic–mass spectrometric method for the determination of isoflavonoids, coumestrol, and lignans in food samples. Anal Biochem. 1996;233(2):169-80.
6. Ross AB, Shepherd MJ, Schüpphaus M, Sinclair V, Alfaro B, Kamal-Eldin A, et al. Alkylresorcinols in cereals and cereal products. Journal of Agricultural and Food Chemistry 2003;51(14):4111-8.
7. Chen Y, Ross AB, Aman P, Kamal-Eldin A. Alkylresorcinols as markers of whole grain wheat and rye in cereal products. Journal of Agricultural and Food chemistry. 2004;52(26):8242-6.
8. Landberg R, Kamal-Eldin A, Andersson A, Vessby B, Åman P. Alkylresorcinols as biomarkers of whole-grain wheat and rye intake: plasma concentration and intake estimated from dietary records. Am J Clin Nutr. 2008;87(4):832-8.
9. Soycan G, Schär MY, Kristek A, Boberska J, Alsharif SNS, Corona G, et al. Composition and content of phenolic acids and avenanthramides in commercial oat products: Are oats an important polyphenol source for consumers? Food Chemistry: X. 2019;3:100047.
10. Pridal AA, Böttger W, Ross AB. Analysis of avenanthramides in oat products and estimation of avenanthramide intake in humans. Food Chem. 2018;253:93-100.
11. Shewry PR, Piironen V, Lampi A-M, Nyström L, Li L, Rakszegi M, et al. Phytochemical and fiber components in oat varieties in the HEALTHGRAIN diversity screen. Journal of Agricultural and Food Chemistry. 2008;56(21):9777-84.
12. Mattila P, Pihlava J-m, Hellström J. Contents of phenolic acids, alkyl- and alkenylresorcinols, and avenanthramides in commercial grain products. Journal of Agricultural and Food Chemistry. 2005;53(21):8290-5.

Additional file 2

Summary of the deconstructed FFQ cereal items used to estimate cereal polyphenol intake in the Melbourne Collaborative Cohort Study

MCCS Item description	Percentage ingredients/contribution (%)	Proxy ingredients (%)[1]
Wheatgerm	wheatgerm (100)	N/A
Muesli	oats (67); oil (8); dried fruit (15); coconut (2); nuts (2); bran (2); honey (2); seeds (2); cinnamon (1)	oats (65), bran (2)
Other breakfast cereals	porridge (10); wheat-based breakfast cereal (40); bran-based breakfast cereal (10); corn-based breakfast cereal (15); rice-based breakfast cereal (15); oat-based breakfast cereal (10)	porridge: oats (100); wheat-based breakfast cereal: wholegrain flour (97); bran-based breakfast cereal: wheat bran (85); corn-based breakfast cereal: wholegrain maize (88); rice-based breakfast cereal: refined rice (91); oat-based breakfast cereal: oats (85).
Rice, boiled (including brown)	white rice (90); brown rice (10)	N/A
Fried rice	white rice (45), pork (27); shrimp (13); spring onion (4);	white rice (45)

**MCCS Item description**	**Percentage ingredients/contribution (%)**	**Proxy ingredients (%)[1]**
	egg+yolks (7); peas (3); oil (2); salt and stock powder	
Mixed dishes with rice	rice (37); chicken stock (37); onion (11); butter (6); parmesan (6); oil (1); herbs (1); garlic (1); white/wholegrain 90/10	rice (40) - white/wholegrain 90/10
White bread, rolls or toast	white bread (100)	N/A
Wholewheat or rye bread, rolls or toast	wholewheat bread (80); rye bread (20)	flour (65)
Fruit bread	wheat flour (47); water (28); dried fruit (18); sugar (6); spices, yeast (1)	white wheat bread (80)
Crackers or crispbreads	wheat flour white/wholewheat (80); oil (10); sugar (3); salt (3); malt extract (1); baking powder (1); yeast (1); other (1); wheat/rye 90/10	white four (40), wholewheat flour (40); wheat/rye 90/10

MCCS Item description	Percentage ingredients/contribution (%)	Proxy ingredients (%)[1]
Sweet biscuits	wheat flour (40); chocolate (20); sugar (20); golden syrup (8); oil (8); cocoa (2); other (2); wheat-based/oat-based (90/10)	Wheat-based: white wheat flour (40); oat based flour/oats (20/15); wheat-based/oat-based (90/10)
Cakes or sweet pastries	wheat flour (42); butter (21); milk (15); sugar (15); eggs (7)	wheat flour (45)
Puddings	wheat flour (18); milk (29); water (23); sugar (13); eggs (10); butter (7).	white wheat flour (20)
Pasta or noodles	pasta/noodles (100)	N/A
Pizza	wheat flour (50); tomatoes (34); mozzarella (7); oil (3); water (2); parmesan (2); garlic (1); salt (1), yeast, sugar, pepper (1)	white wheat flour (50)
Dim sims or spring rolls	cabbage (29); wheat flour (18); beef (18); pork (18); onion (10); soy sauce (2); oil (2); corn flour (2); sugar, salt; pepper (1)	white wheat flour (20)
Pies or savoury pastries	wheat flour (33); water (27); beef (14); margarine/animal fat	white wheat flour (35)

**MCCS Item description**	**Percentage ingredients/contribution (%)**	**Proxy ingredients (%)[1]**
	(14); cheese (3); bacon/pork (3); egg (3); onion (3); salt sugar, other (1)	

N/A = not applicable.

[1] Note: item percentages may vary and may not add to 100% due to rounding.

Additional file 3:

Summary of ferulic acid retention factors (RF)[1] relevant to the MCCS Food Frequency Questionnaire cereal items

Food	Process	Ferulic acid mean RF
**Common wheat, whole grain flour Wheat to bread**	Baked	1.25
**Common wheat, whole grain flour, with bran (6% bran and 10% bran, flour to bread)**	Baked	1.44
**Maize, whole grain (raw corn, yellow and high carotenoid, to tortillas)**	Baked	0.83
**Maize, whole grain (raw corn, yellow and high carotenoid, to corn chips)**	Fried	0.82
**Oats (No food processing found in PED)**	Boiled or baked	1.00
**Rice (raw rice, boiled)**	Boiled	0.62
**Rye, whole grain flour (rye to rye bread baking)**	Baked	1.08

[1] Adapted from RF values in Phenol-Explorer Database v 3.6 [1, 2]

REFERENCES

1. Rothwell, J.A., et al., *Phenol-Explorer 3.0: a major update of the Phenol-Explorer database to incorporate data on the effects of food processing on polyphenol content.* Database (Oxford), 2013. **2013**: p. bat070.
2. Phenol-Explorer Version 3.6. *Phenol-Explorer website*. 2016 [cited 2020; Available from: http://phenol-explorer.eu/search.

Additional file 4

Reporting checklist for observational studies in nutritional epidemiology.

Based on the STROBE-nut guidelines.

Instructions to authors

Complete this checklist by entering the page numbers from your manuscript where readers will find each of the items listed below.

Your article may not currently address all the items on the checklist. Please modify your text to include the missing information. If you are certain that an item does not apply, please write "n/a" and provide a short explanation.

Upload your completed checklist as an extra file when you submit to a journal.

In your methods section, say that you used the STROBE-nutreporting guidelines, and cite them as:

Lachat C, Hawwash D, Ocké MC, Berg C, Forsum E, Hörnell A, Larsson C, Sonestedt E, Wirfält E, Åkesson A, Kolsteren P, Byrnes G, De Keyzer W, Van Camp J, Cade JE, Slimani N, Cevallos M, Egger M, Huybrechts I. Strengthening the Reporting of Observational Studies in Epidemiology-Nutritional Epidemiology (STROBE-nut): An Extension of the STROBE Statement.

		Reporting Item	Page Number
**Title and abstract**			
Title	#1a	Indicate the study's design with a commonly used term in the title or the abstract	1, 3
Abstract	#1b	Provide in the abstract an informative and balanced summary of what was done and what was found	3 - 4

None	#nut-1	State the dietary/nutritional assessment method(s) used in the title or in the abstract.	3
**Introduction**			
Background / rationale	#2	Explain the scientific background and rationale for the investigation being reported	5 - 6
Objectives	#3	State specific objectives, including any prespecified hypotheses	6
**Methods**			
Study design	#4	Present key elements of study design early in the paper	6 - 7
Setting	#5	Describe the setting, locations, and relevant dates, including periods of recruitment, exposure, follow-up, and data collection	6 - 13
Eligibility	#6a	Cohort study: Give the eligibility criteria and the sources and methods of selection of participants. Describe methods of follow-up. Case-control study: Give the eligibility criteria and the sources and methods of case ascertainment and control selection. Give the rationale for the choice of cases and controls. Cross-sectional study: Give the eligibility criteria, and the sources and methods of selection of participants.	6 - 7
None	#6b	Cohort study: For matched studies, give matching criteria and number of exposed and unexposed. Case-control study: For matched studies, give	n/a

		matching criteria and the number of controls per case.	
Variables	#7	Clearly define all outcomes, exposures, predictors, potential confounders, and effect modifiers. Give diagnostic criteria, if applicable	7 - 12
Data sources and measurement	#8	For each variable of interest give sources of data and details of methods of assessment (measurement). Describe comparability of assessment methods if there is more than one group. Give information separately for for exposed and unexposed groups if applicable.	7 - 13, Additional files 1-3
Bias	#9	Describe any efforts to address potential sources of bias	20
Study size	#10	Explain how the study size was arrived at	6 - 7
Quantitative variables	#11	Explain how quantitative variables were handled in the analyses. If applicable, describe which groupings were chosen, and why	8
Statistical methods	#12a	Describe all statistical methods, including those used to control for confounding	

12 - 13

Subgroups and interactions	#12b	Describe any methods used to examine subgroups and interactions	12 - 13
Missing data	#12c	Explain how missing data were addressed	9 - 10

Loss to follow up	#12d	Cohort study: if applicable, explain how loss to follow-up was addressed. Case-control study: if applicable, explain how matching of cases and controls was addressed. Cross-sectional study: if applicable, describe analytical methods taking account of sampling strategy.	6
Sensitivity analysis	#12e	Describe any sensitivity analyses	
n/a			
None	#nut-5	Describe any characteristics of the study settings that might affect the dietary intake or nutritional status of the participants, if applicable.	6
None	#nut-6	Report any particular dietary, physiologic, or nutritional characteristics that were considered when selecting the target population.	6 - 7
None	#nut-7.1	Clearly define foods, food groups, nutrients, or other food components (e.g., preparation method, taxonomical descriptors, classification, chemical form).	8 – 11, Additional files 1-3
None	#nut-7.2	When calculating dietary patterns, describe the methods to obtain them and their nutritional properties.	7
None	#nut-8.1	Describe the dietary assessment method(s) (e.g., portion size estimation, number of days and items recorded, how it was developed and administered, and how quality was	7

		ensured); report if and how supplement intake was assessed.	
None	#nut-8.2	Describe and justify food-composition data used; explain the procedure to match food composition with consumption data; describe the use of conversion factors, if applicable	7 – 12
None	#nut-8.3	Describe the nutrient requirements, recommendations, or dietary guidelines and the evaluation approach used to compare intake with the dietary reference values, if applicable	n/a There are no DRVs for polyphenols
None	#nut-8.4	When using nutritional biomarkers, additionally use the STROBE-ME; report the type of biomarkers used and usefulness as dietary exposure markers	n/a Biomarkers not used
None	#nut-8.5	Describe the assessment of nondietary data (e.g., nutritional status and influencing factors) and timing of the assessment of these variables in relation to dietary assessment	6 – 7
None	#nut-8.6	Report on the validity of the dietary or nutritional assessment methods and any internal or external validation used in the study, if applicable	7
None	#nut-9	Report how bias in dietary or nutritional assessment was addressed (e.g., misreporting, changes in habits as a result of being measured, data imputation from other sources).	8 - 10, 19 – 20
None	#nut-11	Explain categorization of dietary/nutritional data (e.g., use of N-	12

		tiles and handling of nonconsumers) and the choice of reference category, if applicable.	
None	#nut-12.1	Describe any statistical method used to combine dietary or nutritional data, if applicable.	12 – 13
None	#nut-12.2	Describe and justify the method for energy adjustments, intake modeling, and use of weighting factors, if applicable	12
None	#nut-12.3	Report any adjustments for measurement error (i.e., from a validity or calibration study).	7
**Results**			
Participants	#13a	Report numbers of individuals at each stage of study—eg numbers potentially eligible, examined for eligibility, confirmed eligible, included in the study, completing follow-up, and analysed. Give information separately for for exposed and unexposed groups if applicable.	6 – 7, 15
Non-participation	#13b	Give reasons for non-participation at each stage	6 – 7, 15
Participant journey	#13c	Consider the use of a flow diagram	
15			
Descriptive data	#14a	Give characteristics of study participants (eg demographic, clinical, social) and information on exposures and potential confounders. Give	14, 30 - 32

		information separately for exposed and unexposed groups if applicable.	
Missing data	#14b	Indicate number of participants with missing data for each variable of interest	
13			
Follow-up time	#14c	Cohort study: Summarise follow-up time (eg, average and total amount)	
6			
Outcome data	#15	Cohort study: report numbers of outcome events or summary measures over time. Case-control study: report numbers in each exposure category, or summary measures of exposure. Cross-sectional study: report numbers of outcome events or summary measures.	13 - 14, 30 - 32
Main results	#16a	Give unadjusted estimates and, if applicable, confounder-adjusted estimates and their precision (eg, 95% confidence interval). Make clear which confounders were adjusted for and why they were included	30 - 32
Category boundaries	#16b	Report category boundaries when continuous variables were categorized	30 - 32
Relative and absolute risks	#16c	If relevant, consider translating estimates of relative risk into absolute risk for a meaningful time period	
n/a			

Other analyses	#17	Report other analyses done—eg analyses of subgroups and interactions, and sensitivity analyses	30 - 32
None	#nut-13	Report the number of individuals excluded on the basis of missing, incomplete, or implausible dietary and nutritional data.	13
None	#nut-14	Give the distribution of participant characteristics across the exposure variables, if applicable; specify if food consumption for the total population or consumers only was used to obtain results	30 - 32
None	#nut-16	Specify if nutrient intakes are reported with or without the inclusion of dietary supplement intake, if applicable.	n/a Dietary supplements not reported
None	#nut-17	Report any sensitivity analysis (e.g., exclusion of misreporters or outliers) and data imputation, if applicable	13
**Discussion**			
Key results	#18	Summarise key results with reference to study objectives	13 - 16
Limitations	#19	Discuss limitations of the study, taking into account sources of potential bias or imprecision. Discuss both direction and magnitude of any potential bias.	19 - 20
Interpretation	#20	Give a cautious overall interpretation considering objectives, limitations, multiplicity of analyses, results from similar studies, and other relevant evidence.	20 - 21

Generalisability	#21	Discuss the generalisability (external validity) of the study results	20 - 21
None	#nut-19	Describe the main limitations of the data sources and assessment methods used and implications for the interpretation of the findings	19 - 21
None	#nut-20	Report the nutritional relevance of the findings, given the complexity of diet or nutrition as an exposure.	20 - 21
**Other Information**			
Funding	#22	Give the source of funding and the role of the funders for the present study and, if applicable, for the original study on which the present article is based	23
Ethics	#nut-22.1	Describe the procedure for consent and study approval from ethics committee(s).	7, 23
Data statement	#nut-22.2	Provide data collection tools and data as online material or explain how they can be accessed	23

Notes:

- 8: 7 - 13, Additional files 1-3
- 14a: 14, 30 - 32
- 15: 13 - 14, 30 - 32
- nut-7.1: 8 – 11, Additional files 1-3
- nut-8.3: n/a There are no DRVs for polyphenols
- nut-8.4: n/a Biomarkers not used
- nut-9: 8 - 10, 19 – 20

- nut-16: n/a Dietary supplements not reported The STROBE-nut checklist is distributed under the terms of the Creative Commons Attribution License CC-BY. This checklist was completed on 10. August 2022 using https://www.goodreports.org/, a tool made by the EQUATOR Network in collaboration with Penelope.ai
